# Supplementary material for: Are carbon emissions trading and green financial instruments synergistic? -Comprehensive quantitative research based on content analysis
Source: PLoS One. 2024 Mar 7;19(3):e0298601. doi: 10.1371/journal.pone.0298601 (PMC10919601; doi:10.1371/journal.pone.0298601)
Supplement: S1 Data — (ZIP) [file pone.0298601.s001.zip › Data/地级市Z_F工作报告.zip!/╜¡╬≈/╨┬╙α╩╨/└┤╫╘╣1⁄2╓┌║┼í╛┬φ┐╦╩2╛▌═°í┐_2006.docx]

各位代表：

　　 现在，我代表市人民政府作政府工作报告，请各位代表连同《新余市国民经济和社会发展第十一个五年规划纲要（草案）》一并审议，并请市政协委员和其他列席会议的同志提出意见。

　　 一、“十五”和２００５年的工作回顾

　　 “十五”时期是我市经济发展最快、增长质量最好的时期之一，也是城乡建设成就最大、人民得到实惠最多的一个时期。五年来，全市上下坚持以率先崛起为主题，认真实施“一条主线、五篇文章”的总体思路，积极进取，奋发有为，经济社会发展成就瞩目，主要经济指标提前一年以上超额完成“十五”计划预期目标，为加快实现全面小康奠定了坚实基础。

　　 ———五年来，我们始终坚持以工业化为战略核心，国民经济快速发展，综合经济实力跃上新台阶。全市生产总值由６４．９４亿元增加至１７７．３２亿元，财政总收入由７．８５亿元增加到２３．０３亿元，其中地方财政收入由３．７６亿元增加到１０．５３亿元，分别超出“十五”预期目标５４．９％、１３０．３％和８２．２％。累计完成全社会固定资产投资２７３．５７亿元，是“九五”时期的３．７４倍。三次产业结构由２０．５:４３．７:３５．８优化为１２．５:５５．７:３１．８。组建了新余经济开发区，成功实施了“规模以上工业三年翻番”战略，全市规模以上工业增加值、销售收入、利税总额分别比２０００年增长３．７１倍、２．８９倍和１．９０倍。民营工业销售收入占全市规模以上工业的比重由５．５％提升到２２．５％。全市工业化速率达到４５．４％，比２０００年提高７．４个百分点。农业基础地位得到加强，农村税赋政策实现了由“少取”向“多予”的转变，农业经济实现了由以农为主向农业与林牧渔业并驾齐驱的转变，农民收入实现了由务农收入为主向经营性、务工性收入为主的转变。四大上网工程全面实施，商贸市场体系更加完善，新兴第三产业加快发展。全市金融机构年末存款余额由６７．０１亿元增加到１５９．０５亿元，贷款余额由５５．０３亿元增加到１００．６５亿元，年均分别增长１８．９％和１２．８％。仙女湖景区被列为国家重点风景名胜区，全市旅游总收入年均递增２１．２％。

　　 ———五年来，我们始终坚持以城市化为主线，城乡面貌发生深刻变化，建设区域中心城市实现新跨越。修编了城市总规划，提出并着手实施了“两江四岸、组团开发”城市建设总体构想，启动了经济开发区新城区、孔目江新城区、仙女湖旅游城建设，初步奠定了一城五区的建设格局。切实加强重大市政工程建设，仰天岗大道、魁星阁路、天工大道、渝州大桥、新欣大道等城市路桥建成通车，城市防洪工程、污水处理厂、生活垃圾填埋场等市政公用设施相继建设，一批现代化住宅小区陆续兴建。配合推进赣粤、沪瑞高速公路建设和浙赣铁路电气化改造，新余区位优势日趋凸显。治理“两江五湖”，高起点组织了沿江路旧城改造、城区道路“三位一体”改造和“穿衣戴帽”示范工程；启动“三城”创建，重点对“城中村”？？？四眼井等居民小区及城市背街小巷实施了综合整治，先后荣获全国市容环境综合整治优秀城市、全国道路畅通工程样板城市、全国绿化先进城市、全国卫生城市等称号。创新城市经营理念，组建城投公司和土地储备中心，五年共筹措城建资金２１．４９亿元。突出抓好“十镇百村”建设，小康文明村镇建设走在全省前列，农村人居环境明显改善，率先在全省基本实现村村通水泥公路、客车、广播电视、程控电话。城镇化速率达到４８．０％，提高９个百分点。

　　 ———五年来，我们始终坚持以体制机制创新为强动力，改革攻坚取得重大突破，开放型经济发展跨入新阶段。坚持以改革总揽全局，组建了国有资产经营公司和产权交易中心，建立了国有资产三级营运架构，在全省首先实行了行政事业单位国有资产统一经营管理；基本完成了市属工商企业改组改制。率先在全省推行了公安警务体制改革和事业单位人事制度改革、农村信用社改革试点，农村税费改革、乡镇机构改革、林业产权制度改革顺利推进，国家出台的计划、投资、财税等体制改革得到落实。全力实施大开放主战略，五年外贸出口总额达１１９０５万美元，累计利用外资２００４２万美元，实际引进内资１５７亿元，分别相当于“九五”时期的１．５５倍、３．１５倍和１４倍。连年被评为全省横向经济协作先进市。

　　 ———五年来，我们始终坚持以构建和谐社会为目标，人民生活进一步改善，社会保障事业得到新发展。城镇居民人均可支配收入由５６０４元增加到９０５９元，农民人均纯收入由２４９１元增加到３８４０元，年均分别增长１０．１％和９．０％。居民购买能力明显提高，城乡居民储蓄存款余额由５０．１１亿元增加到１０９．４６亿元，社会消费品零售总额由２６．０４亿元增加到４６．５３亿元。城乡就业和社会保障体系更加完善，累计提供就业岗位１８万个，对外劳务输出１６万人；新增城镇从业人员８５２０２人，下岗失业人员再就业１８９１３人，安置困难群体再就业４０２０人。我市被列为全国１００个创业培训示范试点城市之一。率先在全省实现离退休人员养老金发放率和社会化发放率、下岗职工基本生活费发放率、社会保险费代缴率及失业保险金发放率等五个１００％。完善了城乡特困群众救助体系，城市低保对象基本做到应保尽保，农村低保试点被列为全省示范市。

　　 ———五年来，我们始终坚持以经济社会统筹发展为方略，民主法制建设进一步加强，各项社会事业开创新局面。完成了第一次全市经济普查。实施科教兴市战略取得明显成绩，累计实施各类科技项目４２７项，比“九五”时期增长２．１倍；启动了省市共建科普示范城工程，科技进步综合水平晋升全省第四。基础教育加快发展，素质教育全面推进，农村教育走在全省前列。率先在全省启动普及高中阶段教育工程，全市高考成绩连续四年居全省前茅。民办职业教育被誉为“新余现象”，２００５年我市被评为全国设区市中惟一的职业教育先进单位，并成功承办了全国首次民办中等职业教育工作经验交流会。一批优秀广电、文艺作品在全国、全省获奖，组织了一批大型系列群众文艺展演活动。维护食品、药品安全和公共卫生安全进一步加强，抗击“非典”斗争取得阶段性胜利，新型农村合作医疗试点走在全省前列。筹备十二届省运会有序推进，成功承办了第五届农运会乒乓球比赛和中日韩埠际“妈妈队”乒乓球赛。反腐倡廉和依法行政得到加强，建设法治政府成？？明显。我市成为全省惟一跻身中国城市竞争力排行榜“诚信政府”前３０名的设区市。创建“平安新余”扎实开展，安全生产平稳推进，连年荣获全省社会治安综合治理先进市称号，并首次获得全国社会治安综合治理“长安杯”。国防动员和人防、民兵预备役继续加强，连续四届荣获全国“双拥模范城”称号。质监工作被评为全国质量兴市先进市，计划生育综合考评连年荣获全省第一，妇儿、信访、环保工作保持全国、全省领先。金融、邮政、通讯、石油、烟草、盐业、医药等专营性行业实现新发展，工商、税务、公路、稽征、气象、地震、无线电、新闻出版等各项事业迈开新步伐，审计、物价、机构编制、外事侨务、民族宗教、档案、老龄、残联、地方志、驻外机构、关心下一代等各项工作都有新进步。

　　 各位代表，过去的五年，我们经受了“非典”、严重干旱的严峻考验，克服了体制转轨、经济转型中的重重困难，全市改革开放和现代化建设在新的起点上阔步前进。成绩来之不易！这是全市人民在省委、省政府和市委的正确领导下开拓创新、团结拼搏的结果，是人大依法监督、政协民主监督和积极参与支持的结果，是驻市中央省属单位、部队、武警、各民主党派、工商联、人民团体、离退休老同志和台湾同胞、港澳同胞、华人华侨大力支持的结果。在此，我代表市人民政府，向所有关心、支持新余发展的同志们、朋友们表示衷心的感谢和崇高的敬意！

　　 ２００５年，全市上下坚持以科学发展观为指导，全力实施十大重点工程，国民经济继续保持高位增长。全市生产总值增长１７．１％，全社会固定资产投资增长３０．０％，社会消费品零售总额增长１６．１％，财政总收入和地方财政收入分别增长２６．７％和３９．６％。

　　 一是决战工业５００亿开局良好，工业经济效益再攀新高。全市规模以上工业增加值６１．８０亿元、产品销售收入２３８．４６亿元、利税总额１４．０６亿元，分别增长３０．９％、４３．５％和１８．２％。工业经济效益综合指数达１４５．８％，提高１８．１个百分点。优强企业主导工业增长格局得到加强。全市销售收入过亿元的工业企业达２８家，增加１８家；市属规模以上工业增加值增长７９．６％，增幅居全省第一。园区工业加速成为工业经济的重要增长极，经济开发区实现工业增加值、产品销售收入、财政总收入分别增长１０５．８％、１５８．２％和７３．７％；分宜工业园和渝水良山工业园分别实现销售收入２．８亿元和７．３亿元。

　　 二是一批重点项目相继实施，城市功能日趋完善。编制了四个片区控规和八个专项规划。启动了孔目江新城区建设工程，核心区“三通一平”和上游段景观坝建设抓紧进行。“七路三桥”和经济开发区、仙女湖旅游城路网建设加快推进。白云水库建设准备工作有序实施，城南供水管网改造如期竣工，仙女湖旅游城供水管网建设和城东供气工程开始启动。１３条背街小巷得到整治。文化健身广场、劳动力就业大市场、商检大楼正在抓紧建设。铁路二通道建设进展顺利，武吉高速公路征地拆迁正在进行。

　　 三是农村经济稳健发展，农业产业化程度不断提高。全面落实各项惠农政策，维护粮食安全得到加强。农业产业化经营加快发展，全市新增省级农业产业化龙头企业４家，新开发新余蜜桔６２００亩，各类养殖专业户超过４０００户，农民人均可从中获得收入３７１．４元。袁惠渠干渠续建配套与节水改造工程顺利实施，６１座病险水？？除险加固全部动工。十万农民培训工程加快实施，全年培训农民３．５万人。

　　 四是招商引资工作扎实推进，开放型经济加快发展。全年新签内资项目２９７个、外资项目３１个，实际引进内资５１．３３亿元、外资７１６３万美元，分别增长１６．４％和３８．９ ％。工业项目居主导地位，新引进５０００万元以上工业项目２８个，同比增加１４个；实际进资２２．４４亿元，占工业项目进资总数的６０．０％。对外贸易保持高速增长。全市外贸进出口总额１７３５２万美元，增长２０．６％，其中出口４２２９万美元，增长８６．２％。

　　五是第三产业加快繁荣，大市场、大商贸、大流通格局进一步形成。中心城区商贸体系和网点布局进一步完善。城北人民广场改造基本竣工，中国轻纺城现代物流中心、财富步行街、洪客隆商业步行街、抱石商业步行街、中国商贸城加紧建设。仙女湖景区创建国家４Ａ级景区成功获批。春龙国际大酒店等餐饮娱乐设施开工在建。全市银行业实现账面经营收益１．３８亿元，增长２７％。

　　成绩鼓舞人心，形势催人奋进。我们也清醒地看到加快发展中存在的困难和问题：支柱产业单一，产业层次偏低，培育优势产业集群任重而道远；城市功能不够健全，规划体系不够完善，城市文化品位和管理水平亟待提升；县域经济不发达，产业特色不鲜明，“三农”问题依然突出；城乡居民就业、增收压力加大，社会不稳定等因素依然存在；机关工作作风和政府效能建设有待改进，投资环境有待进一步优化。对此，我们将切实增强使命感和责任感，发扬成绩，克服不足，以更加扎实的作风和永久奋斗的精神，努力把政府工作做得更好。

　　二、“十一五”时期的奋斗目标和战略举措

　　未来五年，是我市建设现代区域中心城市、加快实现全面小康的关键五年。在推进新余率先崛起中，我们必须更加注重科学发展，更加注重结构创新，更加注重城乡统筹，更加注重改革开放，更加注重社会和谐，正确处理好速度与效益、效率与公平、当前发展与长远发展的关系，努力实现经济效益、社会效益和环境效益的有机统一，使全社会成员共享改革发展的成果。

　　今后五年工作的总体要求是：高举邓小平理论和“三个代表”重要思想伟大旗帜，全面贯彻落实科学发展观，以加快工业崛起为核心，以打造宜居城市为载体，以推进城乡一体化为着力点，以体制机制创新为主动力，大力实施开放带动、科教兴市和可持续发展战略，努力把新余建设成为经济繁荣、社会和谐、环境优美、人民富裕的现代区域中心城市，健步跨入宽裕型小康社会。

　　全市经济社会发展的总体奋斗目标是： ⑴增创城市综合实力新优势，加快建立对接省会、辐射周边、区域互动的中心城市发展新格局。到２０１０年，全市实现生产总值３６０亿元，财政总收入６０亿元，其中地方财政收入３０亿元，均比２００５年增长１倍以上。全社会固定资产投资累计完成８００亿元，实现社会消费品零售总额９０亿元，外贸出口达到３亿美元。人均生产总值力争突破４０００美元。城镇居民人均可支配收入１６０００元，农民人均纯收入６２００元，年均分别增长１２％和１０％。

　　 ⑵增创体制机制新优势，加快建立对接“长珠闽”、融入全球化的开放带动型经济发展新格局。实施对接战略，在观念、政策、体制、产业、要素市场等方面加快与国际惯例接轨。国有工商企业改组全面完成，以非公有制为主体、股份制改造为基本模式的多元产权结构初步确立。

　　 ⑶增创工业经济发展新优势，加快建立以优势产业集群为依托、以现代制造业为支撑的工业发展新格局。到２０１０年，全市实现工业增加值２００亿元，利税总额８０亿元。中国南方建材精品基地基本建成，经济开发区和工业园区成为工业经济的增长极。基本形成以钢铁为龙头的多元支柱产业体系。

　　 ⑷增创宜居城市新优势，加快建立一城五区、组团开发的城市建设新格局。建设精品城市和品牌城市取得重大突破，主城区达到５５平方公里和４５万人口，城市化速率达到５５％以上。人居环境１３项指标全部达到“中国人居环境奖”标准。全面建成国家园林城市、国家卫生城市和全国文明城市。

　　 ⑸增创农业和农村发展新优势，加快建立城乡联动、统筹发展的城乡一体化新格局。按照生产发展、生活宽裕、乡风文明、村容整洁、管理民主的要求，全面推进社会主义新农村建设，建立城乡统筹发展的长效机制，逐步缩小城乡差别。

　　 ⑹增创和谐平安新余新优势，加快建立以人为本、关注民生、社会事业协调发展的新格局。全面构建城乡统筹发展的就业和社会保障体系，区域性劳务输出中心基本建立，城乡居民人人享有社会保障，城镇人口登记失业率控制在４％以下。公共安全体系更加健全，社会治安更加稳定。各项社会事业明显进步，全国职业教育示范市、全省科普教育示范城全面建成，计划生育国策全面落实，城乡医疗卫生服务体系日趋完善，人人享有初级保健。建设生态文化城市取得重大进展，城乡生态环境得到明显改善。

　　为此，我们将重点组织实施“十大建设规划”：⑴工业经济新体系建设规划。按照“做强一个龙头、做大三个板块”的要求，全力支持新钢公司实施第三期技改工程，建设中国南方建材精品基地；大力发展园区经济，基本形成园区工业与其它工业齐头并进的发展格局；实施高新技术产业化和传统产业的现代化改造，积极扶持１００户销售收入过亿元的重点企业，加快培育一批优势企业集群。

　　 ⑵新农村建设规划。重点以十大优质粮食基地建设为支撑，建立市级粮食储备制度，确保粮食总产稳定在１０亿斤水平；以十大现代农业示范园区建设为载体，壮大草食畜禽、花卉苗木、优质苎麻、新余蜜桔等四大产业，发展中药材基地，加快构建以特色产业开发主导农村经济发展的新格局；以培育金融、科技、农机、流通、农副产品安全质量检测和标准化生产等五大服务体系为依托，抓好龙头企业和合作经济组织建设，大力发展农业产业化；以“十镇百村”建设为着力点，实施组组通公路和村村通信息、沼气工程，搞好村镇改水、改厕、改路和电网改造、旧村改造，高标准抓好小康文明村镇建设；以 “十万农民培训工程”为抓手，大力培育现代新农民，加速农民向城镇居民的转变。实施乡镇崛起行动计划，加快建成一批财政收入过亿元的经济重镇和财政收入超千万元的经济强镇。

　　 ⑶精品城市建设规划。坚持以强化动力配置为重点，整体推进城西旅游休闲区和城南仙来物流区建设，科学构建五位一体的城市格局；以十大精品片区建设为重点，全面推进旧城改造和城市建筑风格化改造；以路桥、管网、绿地、文化生活设施等四大体系建设为重点，加快基础设施建设，不断完善城市功能；以十大标志性建筑群建设为重点，大力实施标志性设施建设工程，整体提升城市形象。

　　 ⑷经济一体化建设规划。大力发展外源型经济，加快构建面向沿海的产业对接基地、产业配套基地、原材料供应基地和区域性劳务输出基地；深化各项改革，全面完善政策法规、金融服务、信息网络、人力资源、物流服务、贸易服务、产权交易、口岸服务、技术标准和社会保障等十大体系；适度超前搞好交通、能源、信息等重点建设，切实增强新余的对接功能。

　　 ⑸现代职业教育示范市建设规划。按照扩大规模、提升层次、凸显特色、培育品牌的要求，切实抓好示范性职业院校和示范性专业建设，全面推行“一校一特色”的办学模式，兴建全国一流的区域综合性实习实训基地，建成现代职业教育片区，适时组建职业教育集团，着力打造独具特色的新余职业教育品牌，加快形成以高职本科为龙头、高职专科为主体的办学格局。力争全市职业院校在校学生达到１５万人以上规模，努力把新余建成江南知名的高级技能型人才培训输出基地。

　　 ⑹区域休闲中心建设规划。按照打造中国情爱圣地的要求，以创建中国优秀旅游城市为载体，以提升景区服务功能为依托，实施“情山爱水”主题整体开发，高起点建设东方丽人城等一批大型旅游项目和旅游主题公园，加快建成仙女湖旅游城。以打造城市亮点、市民乐点、游人看点为目标，全面完成抱石公园改造。

　　 ⑺现代物流体系建设规划。着眼于构架与中心城市相适应的商贸物流体系，发展新型业态，加紧兴建一批大型综合市场、专业市场和购物超市；整体改造城市闹区，加快建成一个市级商业中心、四个商业闹区；实施流程再造，完成新亚新商城和商贸流通企业改制重组，加快发展第三方物流；突出仙来新区综合物流片区和经济开发区物流带建设，以货运配载为纽带，加快建成赣西最大的多功能现代物流中心。

　　 ⑻绿色生态新余建设规划。启动重点库区扩容和重大水利工程，完成白云水库建设和城乡居民饮水改造，搞好仙女湖、袁河、孔目江水体保护，着力构建城乡安全供水体系。规划实施一批重大环境保护工程，突出重点工业污染源集中整治，建设城市垃圾、污水收集处理系统，全面完成袁河及袁惠渠综合整治。抓好城市生态走廊和大型绿地建设，重点实施以环城绿色生态圈、城市进出口景观带和“两江一湖”园林绿地系统建设为主的生态改善工程；加强渝水区经济林、分宜县生态林、仙女湖生态公益林建设，推进边远山区和重点林区退耕还林、移民封山工作。

　　 ⑼城乡就业与社会保障体系建设规划。着眼于建立公平的社会保障体系，抓好就业大市场建设，全面构建集就业培训、社会保障、技能鉴定、人力资源开发于一体的区域性中心市场；完善养老、医疗、失业、工伤、生育五项保险制度，构建五保合一的社会保险管理服务体系；实施 “十助”工程，完善农村“五保”和城镇“三无”对象集中供养制度，全面推行新型农村合作医疗，基本建成融各类救助为一体、城乡统筹发展的社会救助体系。

　　 ⑽和谐平安新余建设规划。实施“信用新余”建设工程，全面推进政府信用、金融信用、工商信用建设，营造全社会诚实守信的信用环境。健全公共安全预警机制和应急处置机制，建立完善安全生产长效运行机制，全方位推进公共安全体系建设。完成金盾工程二期建设任务，健全社区平安保障体系，强化社会治安防控机制，确保社会治安综合治理继续保持全国先进。

　　三、２００６年的主要任务

　　今年是“十一五”规划的开局之年。做好今年的工作，意义尤为重要。今年经济社会发展的主要预期目标是：生产总值增长１５％，力争达到１６％；全社会固定资产投资增长２５％，力争达到３０％以上；社会消费品零售总额增长１４％；财政总收入、地方财政收入分别增长１７％和１８％。城镇居民人均可支配收入增长１２％，农民人均纯收入增长１０％。人口生产控制在省下达指标内。

　　㈠决战工业５００亿工程，大力培育优势产业集群

　　继续实施“工业百亿技改”工程，重点抓好江西赛维多晶硅、分宜海螺日产５０００吨水泥熟料、良山钢管Φ２１９ｍｍ钢管、新余华源高档棉涤包芯技改、众一集团１００万吨球团工程等１３个重点新扩改项目，加快柳新工业园、飞宇音响、彬晖服饰、上扬软件等２８个重点续建项目建设，确保全年完成工业技改投入４２．５亿元。强势推进“工业双百计划”，支持永盛工贸等２０户民营骨干企业加大技改力度，扶持新华、华源地毯等重点企业加快发展。实施“以质取胜”战略，加快培育一批名牌产品。发展循环经济，鼓励企业循环式生产，推动产业循环式组合，致力打造资源节约型、环境友好型工业体系。启动制造业信息化工程，力争三年内全面完成重点企业信息化改造。

　　积极推进新钢与新余工业资源的优势整合，全力支持以３００万吨薄板工程为重点的新钢三期技改和厂容厂貌改造。抓好新电２×６０万千瓦改扩建申报。配合做好分电２０万千瓦机组改造和第二期３０万千瓦机组开工建设。协助抓好花矿２×５万千瓦机组改扩建工程。支持赣西供电公司３个变电站新建和城市配电网技术改造。支持省煤炭集团整合开发新余煤炭资源。协助做好江西二化破产、江西钢丝厂债转股工作。支持石油公司油库搬迁和浙赣铁路电气化改造。

　　坚持举全市之力支持经济开发区发展，抓好新城北大道等１５条道路建设，完成渝东大道、新欣南大道改造等工程，健全区内水、电、气管网，加快区内排污管网与市污水处理厂的对接；推进春龙二期、华通电工城、泉州信诚等工业项目建设，加快引进开工一批重点工业项目，确保全年新引进内资１５亿元、外资３０００万美元，新建厂房５０万平方米，实现规模以上工业销售收入６０亿元。继续抓好分宜工业园、渝水良山工业园建设，培植下村、双林等一批特色产业重镇，扶持壮大县域工业。

　　构建政策、融资、技术、培训服务体系，大力发展民营经济。落实加快中小（民营）企业发展的１０条措施，全面赋予民营经济国民待遇。充实担保基金，增强担保中心担保功能；协调金融机构优化信贷结构、扩大信贷规模，探索建立代理投资公司，重点扶持一批优强民营企业。组建民营科技企业协会，抓好生产力促进中心、科技示范基地和产学研基地建设，高标准建成一批民营经济孵化基地。健全民营经济各类行业协会，抓好民营企业家队伍建设和员工培训，提升民营经济从业人员自我创新能力。

　　㈡积极倡导以人为本的城市理念，着力打造一流的人居环境

　　健全规划体系，完成总规报批，全面推进分区规划和专项规划编订，抓好城区重点地段和主干道控制性、修建性详规编制。今年重点编订仙来西大道、抱石大道、仙女湖河下片区和经济开发区东部、北部片区等控制性详规。强化规划管理，建立城区一体、公众参与、专家论证与政府决策相结合的规划管理体制。

　　完善城市基础设施，突出抓好以长青南路立交桥为重点的城区“七路三桥”建设和劳动路立交桥拓宽工程，开工长林北路、中山北路、茶山路、白竹路二期工程等道路。继续推进城区供水、燃气管网改造，加快城市防洪工程？？大型停车场、污水处理厂等市政公用设施建设，新建第四水厂、城东燃气厂。抓好城市精品建设，兴建赣西电力调度指挥中心、执法大厦等高层建筑群。启动仙来新区建设，配套推进袁河南岸项目开发。以暨阳核心区为突破口，加快孔目江新城区建设。

　　推进“三城”创建，继续抓好背街小巷、城乡结合部综合整治。严格绿线管制，抓紧城郊水源涵养林和环城绿化带建设，规划大型休憩广场，启动小游园、小花园工程，加快孔目江新城核心区景观绿地和城市进出口道路景观带建设，高标准建设国家园林城市。启动城南旧城整体改造，分步推进“城中村”改造。今年重点改造四眼井、廖家江等片区。着力打造春龙、奥克斯等一批现代化精品住宅小区。

　　严格城市管理，健全城市长效管理机制，全面推行城市管理综合执法。调整社区规模，完善街道、居委会、社区管理服务职能，探索建立物业管理公司、居委会、社区服务“三位一体”的新型小区管理体制，健全城区四级管理网络。组建水务集团、燃气集团、巴士集团，推进环卫、市政、园林“管保”、“管干”分离，加快城市公用事业市场化步伐。加强规划执法，严格设计审批，全面整治城市违章建筑；强化“五车”整治，完善公交线路，切实加强城市交通循环体系建设；严格全封闭文明施工，实行渣土密闭装运，全面推行小区垃圾袋装化，着力解决好乱搭乱建、乱停乱放、现场搅拌等问题。

　　㈢高度重视“三农”工作，统筹推进社会主义新农村建设

　　以农民增收为核心，加快农业经济结构战略性调整。坚持“多予少取放活”的方针，全面落实各项惠农政策。建立良山、南安、新溪、钤山四大优质稻生产基地，推广订单生产，确保粮食安全。实施“一村一品”工程，大力发展新余蜜桔、优质苎麻，抓好“菜篮子”建设，扩大花卉苗木、中药材、早熟梨、葡萄、冬枣、葛根等特色种植。启动十大现代农业示范基地和１００个生态养殖小区建设，精心实施一批重大农业项目，加速推进优势农产品产业带开发。

　　以完善服务体系为突破口，大力发展农业产业化。实施农产品加工推进工程，用足用好省农发行３０亿元信用贷款，扶持一批优强龙头企业。高度重视农村合作经济组织建设，重点培植生猪、果业、水产、农机等行业协会。着眼于建立现代农业服务新体系，继续实施“无公害食品行动计划”，高标准建设万亩无公害蔬菜等一批无公害农产品基地；抓好市农村合作银行组建，加快建立农业特色产业开发资金市场筹集、滚动投入的新机制；实施“良种良法工程”，全面推行农业机械化生产；强化农副产品质量认证、检验检测和动植物重大疫病防控体系，健全市、县两级质检机构。继续实施十万农民培训工程，力争全年培训农民３．５万人次。

　　以“十镇百村”为重点，全力推进小康文明村镇建设。坚持因地制宜、突出特色，完善村镇规划体系，适度超前搞好“十镇百村”规划。抓好生态沼气建设，完善村镇路网体系，启动农村第二期３万人安全饮水改造，力争三年内全面完成农村改水、改厕、改路、电网改造和村村通信息、沼气工程。整治农村脏、乱、差，分步推进旧村改造、移民并村。

　　以提高农业综合生产能力为目标，切实加强农田水利基本建设。抓紧编制全市水资源综合规划和农田水利基本建设规划。加大袁惠渠和中型水库灌区改造力度，全面完成龙门口和３０座小型病险水库除险加固任务。抓好３万亩中低产田改造。全面完成退耕还林和日本政府贷款？？西造林项目年度任务，力争启动生态公益林补偿项目，加快建设笋竹两用林、南方红豆杉、工业原料林、高效生态型油茶等十大林业示范基地。

　　㈣加快发展第三产业，不断繁荣城乡经济

　　继续实施现代物流体系建设工程，大力发展新型业态，加快繁荣中国商贸城、九鼎汽车大市场等大型专业市场，抓紧洪客隆、抱石商业步行街建设；实施“农改超”工程，新建聚贤苑粮油批发交易中心；加快发展钢铁、水泥、粮食等加工包装储运型第三方物流企业，突出抓好鼎晟钢铁物流项目建设，实施城南、城北两个汽车站整体搬迁，全面启动现代物流中心二期工程，加快建设现代物流中心。

　　抓好仙女湖旅游城建设，策划兴建龙府休闲中心、《天工开物》科技园等一批大型旅游项目和主题公园，启动银河特色街建设，兴建一批高档旅游宾馆和大型娱乐设施；继续实施“六个一”工程，搞好景点串线和精品线路打造，打捆做响仙女湖品牌。编制旅游业发展总体规划，高标准实施抱石公园改造，启动仰天岗主题公园开发，规划论证严嵩故里等一批旅游项目。

　　发展楼宇经济，规范培育房地产市场。实施土地储备计划，创新土地经营方式，强化城市中心区土地控制，规划建设一批高档商务楼，积极引进国内外知名企业设立区域性总部、结算中心、研发中心、培训中心和销售代理。实施鼓励住房消费的积极政策，加大住房公积金对购房的支持力度，培育房地产二、三级市场。发展经济适用房，积极推进廉租房建设。加强年度用地计划约束，推行房地产规模开发，健全房地产开发联审联批协调机制，促进房地产业健康发展。

　　改造提升传统服务业，加快发展新兴第三产业。优化餐饮娱乐设施网点布局，培育大众餐饮市场，规范发展文化健身休闲场所，加快建设一批星级酒店和特色风味小吃街、大众美食城。全面推进“数字新余”建设，推行电子商务、电子政务，加快发展信息服务业。发展公证、仲裁事业，培育会计、审计、评估、咨询、律师等中介服务组织，抓好行业协会、商会等自律性组织建设。扩大消费信贷，规范发展金融、保险、证券、期货、信托、担保、典当、拍卖业，创造条件逐步将北湖中路改造成金融一条街。重视发展家政、广告等其它新型服务业。支持邮政、电信、烟草、盐业、电力、石油、图书发行等专营性行业加快发展。

　　㈤繁荣发展外源型经济，全面深化以市场为取向的各项改革

　　大力实施四大战略，加快构建“三外并举”的发展格局。实施主导项目带动战略，全面加强各级、各系统招商项目库建设，督促抓好泉州工业园、甬温工业园等已签约项目的进资、开工；突出产业导向性和高就业型项目，积极推进产业招商和重点项目招商，加快实现钢铁产业链式化招商。实施联动竞争战略，突出闽浙、港台等重点地区和国内外知名企业，强化重点客商资源库建设，加快发展友好合作伙伴、友好城市；突出以商招商、代理招商，发展离岸公司等招商中介组织，强化驻外机构招商功能，加快培育一批重点招商经纪人。实施对接战略，完善外商服务中心，加快海关开关和检验检测机构建设，全面建设项目代办绿色通道；整治投资环境，着力打造投资、金融安全区。实施市场多元化战略，突出抓好出口基地建设，加快培育三路出口大军；搞好横向经济协作，广泛开拓外经业务，推动外经外贸实现新突破。力争全年引进外资７８８０万美元、内资６２．８亿元，分别增长１０％和２２％；外贸出口总额６０００万美元，？？长４２％。

　　继续抓好国有工商企业改制扫尾，统筹推进各项改革。启动新纺破产、市肉联厂清算解散程序，妥善处理好原新纺、长林、前卫、床上用品厂等已改制企业遗留问题；实施电工厂一分为三的设想，帮助信达长林寻求优强合作伙伴，加快江锂新材料公司重组江西锂厂步伐，继续推进县（区）企业改制改组。做好流通企业改制善后工作。基本完成农口企业和房管系统、城建系统企业改制。实施金财工程，深化部门预算、国库集中收付、政府采购和收支两条线改革；认真落实城区税收征管各项政策，实现城区税收统一征管。巩固农村税费改革成果，深化林业三项改革，统筹推进乡镇机构、农村义务教育和县乡财政体制改革。

　　创新国有资产经营模式，健全国有资本营运、监管机制。深化市直行政事业单位国有资产统一经营管理，重组担保中心，搞好行政事业单位资产整合和资产拍租。完善市国资公司、城投公司和新亚新工贸集团法人治理结构，规范授权经营，全力盘活国有资产。重组市属国有独资及控股企业，全面推行国有资本出资人制度。积极推行矿产资源探矿权、采矿权的统一规划、管理和拍卖，整治开发秩序，推进价格改革，加快矿产资源开发规模化、集约化、市场化进程。

　　㈥统筹推进各项社会事业，促进人的全面发展

　　实施农村教科文卫三年行动，发展农村各项社会事业。落实以县为主的义务教育管理体制，加大农村教育投入，优化学校网点布局，统筹推进学校基本建设，力争三年内全面完成学校现有危旧房改造；做好贫困家庭学生“两免一补”工作；抓好现代远程教育工程，确保两年内农村初中普遍建立计算机教室、小学普遍建立卫星地面接收装置。全面推行农村科技特派员制度，抓好青年星火带头人培训工程，启动国家级和省级科技进步先进县（区）创建活动。完善农村文化、广电基础设施。开展创建全省农村卫生工作先进县（区）和农民满意的村卫生室（所）活动，以房屋、设备、技术和管理四配套为目标，加快乡镇卫生院改造，合理设置村级卫生室（所）。加强农村食品卫生监督，实行食品卫生监督城乡一体化管理。继续抓好新型农村合作医疗试点，争取渝水区列为全省新型农村合作医疗试点区。

　　争创全国职业教育示范市和全省科普教育示范城，加快发展教育科技事业。抓紧职教片区建设，支持渝州学院“专升本”和中山学院“升高职”工作，启动市职教中心新校区建设，着力培育一批省级以上示范性职业院校和特色专业，兴办区域综合性职教实训基地，建立民办学校教师交流中心，支持民办职校加快发展，力争职校在校学生达９万人。抓好高专“专升本”工作。继续推进普及高中阶段教育工程，加快优质高中建设，重点实施好市一中新校区二期、市七中异地建校等项目。统筹规划城区小学网点建设，改造一、二、三小，力争两年内基本解决城区小学教育资源不足的问题。实施学术和技术带头人培养计划，抓好重点科技项目实施，加快产学研联合，推进高新技术产业化试点工程，不断增强企业自主创新能力。启动青少年素质教育实践基地建设和青少年科普教育基地、市科技馆立项兴建工作，继续抓好科普示范县、示范社区、示范村建设，高标准建成一批城市科普设施，争取申报列入国家科普示范试点基地。

　　实施健康城市三年行动，建立完善城市公共卫生体系。加强公共卫生信息网络建设，健全疫情报告和应急指挥系统，完善疾病预防控制、应急医疗救治和卫生执法监督体系。今年重点抓好市人民医院新院和市妇幼保健院住院大楼的开工兴建，建成市传染病专科医院、市紧急救援中心和渝水区疾控中心大楼。开展“医院管理年”活动，全面落实解决群众“看病难、看病贵”问题的２０条措施，继续开展打击非法行医专项行动。实施区域卫生规划，加快构建以社区卫生服务为基础的城市医疗卫生服务体系。

　　全力以赴承办好十二届省运会，繁荣发展文化、体育事业。实施大文化战略，切实加大对文化艺术事业的投入，积极推出一批时代精品力作，加快发展文化、体育产业。突出抓好省运会场馆建设、市场筹资、组织接待等各项准备工作，确保文化健身广场８月底交付使用。积极备战省运会，确保实现“金牌保四，总分前移”目标。兴建文化影视城，着手规划市博物馆、图书馆新馆建设，精心举办好省运会开、闭幕式大型文体表演。发展新闻出版事业，加快新余图书城建设。整合有线广播电视站（网）资源，推进广播电视数字化改造。

　　实施可持续发展战略，统筹推进人与自然的和谐发展。兴建市环保监测监察指挥中心，加快袁惠渠水体污染综合治理，集中开展城市噪声防治和餐饮业油烟治理，抓紧治理新钢、二化、城市生活污水等老污染源。保护基本农田，强化土地整理，确保耕地动态平衡。加强出生人口性别比失衡专项整治，突出抓好城市社区计生工作和重点人群计生管理，建立完善人口与计生工作新机制，确保计生工作继续走在全国、全省前列。继续实施好妇女、儿童《纲要》，做好关心下一代工作，支持关爱老年人、残疾人等事业。抓好农业普查和十大重点调查，健全经济运行预警、监测体系。续编《新余市志》。做好外事侨务、对台、档案、民族宗教工作。强化国防教育，搞好国防动员和民兵预备役工作，落实优抚政策，争创全国“双拥”模范城五连冠。

　　㈦切实关心群众利益，加快构建和谐平安新余

　　落实促进全民创业的各项政策，完善以小额贷款、税费减免、社保和培训补贴为支撑的政策扶持体系，健全各级创业指导服务中心，加快培育各类创业主体。实施增加就业岗位工程，抓好再就业基地和非正规劳动组织建设，加快与工业园区用工对接；以解决“零就业”家庭就业和失地农民就业为重点，开发社区服务和公益性就业岗位，做好困难群体就业和再就业援助工作。抓紧就业大市场建设，确保年内完成主体工程，逐步建立免费开放的“公共实训中心”，健全培训、就业、维权“三位一体”的就业服务体系。力争全年新增城镇就业２万人，安置下岗失业人员再就业７０００人，省外劳务输出１６．５万人。

　　健全城镇职工医疗、工伤和生育保险政策体系，完善大病统筹制度，抓好改制破产企业职工和离退休人员参保和接续工作。做好扩保征缴工作，建立事业单位社保制度，尽快将民营经济从业人员、流动人员和失地农民的三项基本保险统一纳入社会保障体系。启动农村低保，探索建立融基本医疗保险、养老保险、最低生活保障和孤寡老人社会赡养于一体的农村社会保障体系。全面建立市、县（区）、街道（乡镇）、社区社会救助机构，健全“十助”体系，着力解决贫困群众在生产、生活、就医、子女入学等方面的实际困难。完善城乡价格监控网络，加强粮食、农资价格监管，切实抓好教育、医药、水电、电信等专项收费检查。

　　全面建立公共安全预警体系和应急救援、社会动员机制，纵深推进平安新余创建。切实加强社会治安综合治理，启动五五普法规划，完成全国城市报？？？与监控系统建设试点任务，强化治安防控体系，构建国家安全人民防线，严厉打击各种犯罪活动；畅通信访渠道，完善法律救助，健全社会利益协调和社会纠纷调处机制，积极预防和妥善处理群体性突发事件。筹建煤矿应急救援指挥中心，抓好高危行业安全生产专项整治。搞好气象、人防、地震、水文、地质监测，提高防灾减灾能力。实施食品、药品放心工程，构建行业自律机制，深入推进食品药品安全信用体系建设。强化消防、防火、交通等安全监管，努力杜绝重特大安全事故。

　　㈧改进政府效能，建设廉洁高效、为民办实事的政府

　　加快政府管理创新，努力建设法治型政府。推进政事、政企、政社分开，做好中介机构与政府脱钩工作，全面完成事业单位改革，加快政府公共事务社会化、市场化改革步伐。贯彻《全面推进依法行政实施纲要》，健全行政执法责任制、执法过错追究制和执法行为评议考核制；落实主办部门责任制，严格规范行政许可和行政审批行为。推进“阳光行政”，全面推行公共政策、部门责任白皮书制度。实施人才战略，加强高素质公务员队伍建设，推进省市共建江西赣中人才市场，完善高层次人才信息库，加快建立鼓励人才脱颖而出的用人机制。

　　加强法制监督，完善社会主义民主政治。积极办理人大代表、政协委员的建议和提案，认真听取民主党派、工商联、无党派人士及工青妇等人民团体的意见，充分发挥人民群众和新闻媒体的监督作用，自觉把政府的行政行为置于人大依法监督、政协民主监督和社会舆论监督之下。推进民主决策，完善政府新闻发布制度，健全人民群众建议征集制度和重大问题调研评估、专家咨询、公示听证、集体决策制度。办好市政府网站和“市长热线”，拓宽反映社情民意的渠道。扩大基层民主，加强民主法制示范村建设，完善村（居）民自治。

　　积极开展“政府效能建设年”活动，全面提升政府效能。建立统一规范的政务超市，扩大行政服务中心服务职能。完善政务投诉中心，统一办理群众投诉。加强行政效能监察，健全“跟踪督查、定期述职”的抓落实制度，定期组织对政府重大工作事项、工作目标和重点项目的督查考评、述职交帐。完善政府为人民群众办实事、办好事制度，每年为群众办好几件实事。

　　开展节约型机关创建活动，加快构建节约型政府。坚持勤俭办社会事业的方针，广泛开展“六个一”节约活动，努力创建节约型机关。加强财政监管，强化硬预算约束，从严控制出国考察、会务、接待、小车费用支出。落实党风廉政建设责任制与惩治和预防腐败体系实施纲要，强化领导干部廉洁自律，坚决查处大要案；抓好经济责任审计和重大投资项目审计；健全政务环境评议评价制度，开展纠风专项治理，从源头上预防和治理腐败。

　　各位代表！回顾过去，我们取得了令人鼓舞的成绩；展望将来，我们必将开创更加辉煌的未来！让我们紧密团结在以胡锦涛同志为总书记的党中央周围，奋力拼搏，扎实苦干，全面完成今年的各项任务，为在全省率先实现全面小康而努力奋斗！
